# Wild edible plant knowledge, distribution and transmission: a case study of the Achí Mayans of Guatemala

**DOI:** 10.1186/s13002-015-0024-4

**Published:** 2015-06-16

**Authors:** Nerea Turreira-García, Ida Theilade, Henrik Meilby, Marten Sørensen

**Affiliations:** Department of Food and Resource Economics, Faculty of Science, University of Copenhagen, Rolighedsvej 25, Frederiksberg, C 1958 Denmark; Department of Plant and Environmental Sciences, Faculty of Science, University of Copenhagen, Thorvaldsensvej 40, Frederiksberg, C 1871 Denmark

**Keywords:** Acculturation, Ethnobotany, Indigenous, Knowledge loss, Local ethnobotanical knowledge (LEK), Traditional ecological knowledge (TEK)

## Abstract

**Background:**

Knowledge about wild edible plants (WEPs) has a high direct-use value. Yet, little is known about factors shaping the distribution and transfer of knowledge of WEPs at global level and there is concern that use of and knowledge about WEPs is decreasing. This study aimed to investigate the distribution, transmission and loss of traditional ecological knowledge (TEK) concerning WEPs used by a Mayan community of Guatemala and to enumerate such plants.

**Methods:**

The case study was carried out in a semi-isolated community where part of the population took refuge in the mountains in 1982–1985 with WEPs as the main source of food. Major variables possibly determining knowledge and therefore investigated were socio-demographic characteristics, distance to and abundance of natural resources and main source of knowledge transmission. A reference list of species was prepared with the help of three key informants. Information about the theoretical dimension of knowledge was gathered through free listing and a questionnaire survey, while practical skills were assessed using a plant identification test with photographs. All villagers older than 7 years participated in the research (n = 62 including key informants).

**Results:**

A total of 44 WEPs were recorded. Theoretical knowledge was unevenly distributed among the population, and a small group including very few informants (n = 3) mentioned, on average, three times more plants than the rest of the population during the free listing. Practical knowledge was more homogeneously distributed, key informants recognising 23 plants on average and the rest of the population 17. Theoretical and practical knowledge increased with age, the latter decreasing in the late phases of life. Knowledge about WEPs was transmitted through relatives in 76% of the cases, which led to increased knowledge of plants and ability to recognise them.

**Conclusions:**

The WEP survey may serve as a reference point and as a useful compilation of knowledge for the community for their current and future generations. This study shows that the elder and the refugees living in the area for longer time know more than others about WEPs. It also shows the important role of knowledge transmission through relatives to preserve TEK.

## Abstracto

**Antecedentes:**

El conocimiento sobre plantas comestibles silvestres, en Inglés *wild edible plants* (WEPs), posee un alto valor de uso directo. A pesar de ello, se sabe poco sobre los factores que determinan la distribución y transferencia del conocimiento de las WEPs a nivel global y se teme que su uso y conocimiento esté disminuyendo. Este estudio de caso tenía como objetivo examinar la distribución, transmisión y pérdida de conocimiento ecológico tradicional (TEK) relativa a las WEPs utilizadas por una comunidad maya de Guatemala y hacer el inventario de dichas plantas.

**Métodos:**

Este estudio de caso se llevó a cabo en una comunidad semiaislada donde parte de la población se refugió en las montañas en 1982–1985, alimentándose mayormente de plantas silvestres. Las principales variables que influyen el conocimiento y, por tanto, las investigadas, fueron características socio-demográficas, la distancia a- y abundancia de- los recursos naturales y la principal fuente de transmisión del conocimiento. La lista de referencia de especies fue elaborada con la ayuda de tres informantes clave. La información sobre la dimensión teórica del conocimiento se obtuvo a través de un listado libre y un cuestionario, mientras que las habilidades prácticas se evaluaron mediante una prueba de reconocimiento de fotografías de plantas. Todos los habitantes mayores de 7 años participaron en la investigación (n = 62, incluyendo los informantes clave).

**Resultados:**

Se registraron un total de 44 WEPs. El conocimiento teórico se distribuía de manera heterogénea entre la población, donde unos pocos informantes (n = 3) mencionaron, en términos medios, tres veces más plantas en el listado libre que el resto de la población. El conocimiento práctico se distribuía de manera más homogénea, donde los informantes clave reconocieron una media de 23 plantas y el resto de la población 17. El conocimiento y las habilidades de identificación aumentaban con la edad, pero las habilidades disminuían en las últimas fases de la vida. El conocimiento acerca de las WEPs se transmitía a través de familiares in el 76% de los casos, que resultaba en mayor conocimiento sobre las plantas y capacidad para reconocerlas.

**Conclusiones:**

El estudio de las WEPs puede servir como un punto de referencia y como una útil recopilación de los conocimientos de la comunidad para las actuales y futuras generaciones. Este estudio muestra que los mayores y refugiados que viven en la zona durante más tiempo saben más sobre las WEPs que otros y la importancia de la transmisión del conocimiento a través de los familiares para preservar el TEK.

**Palabras clave:**

Aculturación, Conocimiento etnobotánico local (LEK), Conocimiento tradicional ecológico (TEK), Etnobotánica, Indígenas, Pérdida de conocimiento

## Background

There is an intrinsic relationship between humans and their environment and between knowledge about and use of natural resources [1-3]. Traditional cultures are deteriorating which leads to a loss of traditional knowledge worldwide [4]. It is also a general trend that knowledge of wild edible plants (WEPs) decreases, due to the appearance of industrial agriculture and modern food industry, associated shifts in dietary habits and preferences, negative perceptions of WEPs, time consumption associated with WEP collection, and lack of interest among younger generations [5-8].

WEPs are defined here as plants growing spontaneously in an area, i.e. without being cultivated, including native species as well as introduced species that have naturalized, and which are ingested as food in the form of solids or liquids [8]. Food medicines are edible plants that are deliberately consumed for medicinal purposes [6]. WEP knowledge possesses high direct-use value, which helps to reduce the need of buying marketed alternatives and helps to achieve food security [7,9,10]. Simultaneously, WEPs serve as dietary supplements or as famine food in times of scarcity [6,7,11-14].

There are two dimensions of ethnobotanical knowledge; a theoretical and a practical. The former refers to the ability to name plants, whilst the latter refers to the skills needed to put the knowledge into practice or to connect the names to the organisms [15,16].

Several factors have been shown to influence knowledge and use of natural resources, including i) distribution of the natural resource, ii) demographic characteristics, iii) residence period, and iv) occupation [17].

Traditional ecological knowledge (TEK) evolves continuously adding lessons from the past to the present [18]. The initial acquisition of knowledge happens through innovation or diffusion [19]. If an area is isolated with long distances to markets and forest product substitutes, the learning of TEK is a necessity [3]. The first steps in transmission of knowledge and skills related to natural resources and their use include familiarization with the resource, observing, playing and helping adults [20,21]. The knowledge, which is maintained, transferred, or exchanged, is the knowledge that has a use or a value [3] or is essential for subsistence [22]. Local knowledge depends on social transmission, through the family or collectively within a community [19,23,24]. The loss or erosion of TEK is often due to changes in social relationships [25], access to new products [26] or exhaustion of the resource [5,7]. Economic activities that are not related to the local environment tend to negatively affect ecological knowledge [17].

There has been a growing interest in WEPs during the last decade. Studies have focused on surveying WEPs and associated genetic resources [27-29], nutritional values and chemical compounds [30-32], and peoples’ use and knowledge of WEPs [7,11,13,33-38]. Ethnobotanical studies have provided basic information on edible plants, diversity of use and knowledge patterns in different parts of the world. However, academic knowledge is limited with regard to factors shaping the distribution and reproduction of knowledge of WEPs at the global level, which vary according to the specific ecological, cultural, historical and socio-economic context [19].

The aim of this study was to document WEPs used and to understand how the theoretical and practical knowledge vary and are reproduced within a community of Achí Mayans in Guatemala. The study seeks to answer the following research questions i) how are WEPs distributed in the landscape and across seasons, which plant part(s) is(are) used, and do they have medicinal uses as well? ii) how is WEP knowledge perceived to be transmitted and how does transmission influence theoretical and practical WEP knowledge? iii) how is the theoretical WEP knowledge distributed and what determines it? and iv) how is the practical WEP knowledge distributed and what determines it?

The study correlates plant distribution, abundance and other characteristics with traditional knowledge. For example, the fact that a plant is abundant or available [39], grows close to residential areas [17,40], and/or is cultivated or has a domesticated relative could influence the probability of knowing and identifying the plant positively. It is also assumed that knowledge transmission through relatives will influence plant knowledge positively [19,23,24]. Gender [3,36,37,41], age [17,24,41], the age × gender interaction factor [7,11], residence period [41] and occupation [17,41] are all expected to shape the distribution of knowledge. Hence, the study is guided by the following seven hypotheses: (1) the transmission of knowledge regarding WEPs is dominated by transmission from relatives, this having a positive influence on WEP knowledge; (2) WEP knowledge increases with age and residence time; (3) WEP knowledge is particularly high for people who have experienced a period where they depended heavily on wild edible plants; (4) women are more knowledgeable on WEPs than men; (5) older women are particularly knowledgeable on WEPs; (6) living in immediate vicinity of places with a high number of WEPs positively influences knowledge on such resources; (7) maintaining close contact with natural resources by working in the fields and/or visiting the mountains increases WEP knowledge.

## Methods

### Study area

**Figure 1:**
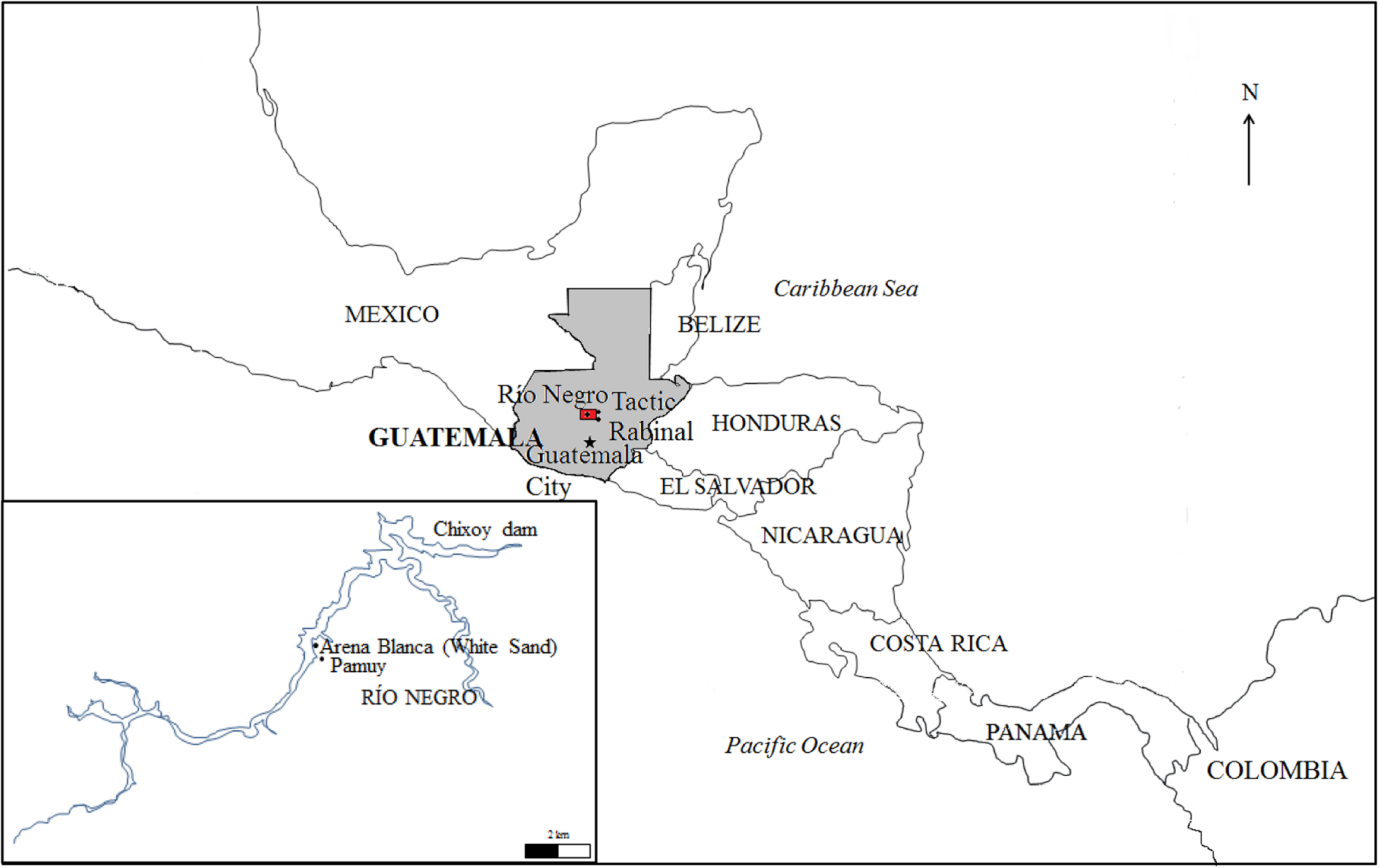
Map of Río Negro in Guatemala and extension of the community. The large map shows the nearest markets, Tactic and Rabinal, and the inserted one the Chixoy dam and the two sub-villages of Río Negro, Arena Blanca and Pamuy.

The research was conducted in Río Negro, an Achí speaking rural community of central Guatemala (15°13′57″N; 90°31′24″W). The village is located at an elevation of 1256–1820 m a.s.l. It belongs to the Plateau climate zone following the Thornthwaite System [42]. Average annual minimum and maximum temperatures (1990–2008) were 18.0°C and 31.5°C and the average annual precipitation was about 1424 mm [43]. Locally, the climate was defined as ‘*tierra caliente*’ (hot land). The most important river basin is river Chixoy or Negro. The vegetation in the highlands is typical of subtropical moist forest (temperate) according to the Holdridge Classification, with dominance of *Pinus oocarpa* Schiede, *Quercus* spp., and *Cupressus lusitanica* Mill., and subtropical dry forest in the lowlands with dominance of *Byrsonima crassifolia* (L.) Kunth and other species of the Leguminosae family [44]. Due to opposition to the construction of the Chixoy dam in the Chixoy river basin in 1982, the inhabitants of Río Negro were accused of being *guerrilleros* and were persecuted. To survive, many people escaped to the mountains where they remained hiding for up to three years. The main type of food available to the refugees was WEPs. Between 1982 and 1985 the survivors were relocated to a military camp. In 1991, three families returned to Río Negro and currently the community includes 17 families and a total of 97 inhabitants. The community is divided into two sub-villages, ‘*Arena Blanca*’ (White sand) hill and ‘*Pamuy*’ valley (Figure 1). The community has remained isolated, with very small patches of fertile land as most fields were flooded following the construction of the dam.

Tourism, fishery and selling handicrafts are the main sources of cash income for villagers while agriculture, fishing and hunting are mainly for subsistence. The subsistence crops are maize, *maicillo* (an unidentified grass), beans and *pepitoria* (ground pumpkin seeds). The commercial crops are beans and *pepitoria*. The crops grow in small agricultural fields in the mountains, under the *milpa* agro-ecological system. The land tenure system is communal. The nearest markets are Rabinal (22 km approx.) and Tactic (32 km), and the inhabitants of Pamuy visit the market every week to sell fish. The inhabitants of Arena Blanca visit the market every three weeks or once a month. There is labour division between genders. All men (>14 years old) cultivate their homegarden and field, go fishing and collect firewood. Most of them are also artisans. All women (>14 years old) are housewives and artisans. All children younger than 14 attend the local school and after school they help their parents at home.

Río Negro was selected for this case study as it offers an opportunity to study a semi-isolated population with two different pasts, including people that were refugees between 1982–1985 and people that were not, and a strong relationship with the surrounding nature.

### Data collection

The fieldwork took place from February to April 2013 and was carried out by the first author. The objectives and methods of the study were explained to and approved by the community in advance. The data collection methods included both qualitative approaches, to get an overview of the study site and population and understand the causes and reasons behind the phenomenon studied, and quantitative approaches, to triangulate and to enable the use of statistical inference. The research followed the ethical principles to be considered when working with indigenous people and their knowledge presented by the ISE (International Society for Ethnobiology) in 2006 [45].

#### Plant reference list

Three key informants participated individually to produce a reference list of WEPs found in the area, using free listing, a questionnaire and a seasonality diagram. All three of them were males and they were 16, 43 and 49 years old, respectively. The key informants were purposefully selected based on their generally known knowledge on the subject.

For the free listing, key informants were asked to name all the wild food plants found in the study area. Based on the reference list a questionnaire was prepared, including the following subjects: plant growth form, common name, Achí name, part of plant used, cultivation of plant or a domesticated relative, who collects it and place of collection (the specific distribution refers to the wild species, not to the cultivated plant or domesticated relative). Furthermore, whether the plant was still used as edible plant or was used only during times when inhabitants lived as refugees in the mountains, method of preparation and if the plant had any medicinal properties was recorded. For medicinal edibles, which part was used, disease(s) treated, recommendations and modes of preparation was documented. A seasonality diagram was constructed recording the relative abundance and availability of each part of the plant throughout the year [46]. To obtain a measure of relative abundance the key informants were asked to place 1–3 dots in each month for each edible part of plants available in that month (● not very abundant; ●● medium abundance; ●●● very abundant).

The answers of the three key informants were crosschecked among them by asking detailed questions about plants that another person had mentioned. If any of the key informants disagreed with an answer of another informant, the plant was not included in the list.

Finally, a community sketch map of 2010 drawn by a villager was updated with the help of a key informant. This helped the first author to become familiar with the area and understand local perceptions of the surrounding nature, boundaries, land uses and location of natural resources.

#### WEP knowledge collection methods

Knowledge is produced and maintained through a dynamic process and evolves continuously; sometimes it is acquired consciously, at other times unconsciously [19]. Hence, this study investigated how knowledge is *perceived* to be acquired, transmitted and lost.

All villagers with an age of more than 7 years who were present in the village at the time of the study (62 informants) were interviewed by the first author. Informants included 52% males, 24 ± 17 years old (mean ± STD), and 48% females, 26 ± 15 years old (mean ± STD). Thirteen temporary migrant workers and 22 children under the threshold age did not participate in the study. Interviews were conducted in Spanish, except in two cases where an interpreter proficient in the Achí language was needed. Achí names were transcribed using a dictionary [47]. Interviews were done individually at the house of the researcher. When this was not possible informants were interviewed outside their own houses. In such cases, members of the same family were interviewed on different days to avoid bias. Children were interviewed at the school individually. The interview was based on a socio-demographic questionnaire (date, name, age, gender, ethnicity, marital status, occupation, first and second language, residence time in the village, current residence place and previous residence place if any). Informants were asked to list all the food plants they knew were growing spontaneously in the study area. This method has been used by several authors to study the intellectual dimension of ethnobotanical knowledge [25,35,39,48]. If plants new to the reference list were mentioned, specific information was recorded and cross-checked with the key informants. The practical skills were assessed using a plant identification test based on photographs [33,49], where a plant was considered as recognised if the informant could state the name of it. The plant photographs used in the plant identification test were obtained from the internet. For six of the local WEPs a photograph was not available and these species were therefore not included in the test. The plants included were based on the list of plants prepared by key informants. New plants that were mentioned later by informants were not included (n = 17). This resulted in 21 plants found in the plant reference list and 7 other plants initially claimed to be wild, such as *Ayote* (*Cucurbita argyrosperma* hort. ex L.H. Bailey). The informants were also asked from where and whom they learned of plants that they recognised.

Semi-structured interviews were conducted with some of the adults to get an insight regarding sensitive topics [46] such as the time of mountain refuge (only the people that had experienced it), migration, differences between genders and daily life.

#### Forest walk and herbarium compilation

The community sketch map and the information from the seasonality diagram were used to design a forest walk to collect WEPs available at the time of the survey. The forest walk was done with the person who mentioned most plants in the free listing exercise. The walk included paths surrounding the households and the trail from the community and up to the mountain fields. Thirteen WEPs were collected, pressed and dried. Photos of common species that were available were taken (n = 11). CITES listed species were not collected (n = 3), instead photos were taken.

### Data analysis

The collected plants of the plant reference list were identified by the first author and botanist David Mendieta, an expert in Guatemalan plants at the herbarium of the Agronomy Faculty, University San Carlos of Guatemala (USAC), where the specimens are stored. Species that were not collected were identified by contrasting photographs taken by the first author (when available), local names, Spanish names, descriptions given by the key informants and distribution of the plant with the information found in *Flora of Guatemala* by Standley and Steyermark (1946–1976) vol. 24, parts I–XIII [50]. The latest accepted nomenclature was checked in http://www.theplantlist.org/.

The data was analysed using SAS/STAT software, Version 9.2 [51]. A Chi-square independence/homogeneity test was carried out to identify variables (Table 1) that show dependency with the two spheres of knowledge, theoretical and practical knowledge. Theoretical knowledge was analysed using a binary variable that takes a value of 1 when the number of plants listed by an informant was >7.15, which was the average number of plants mentioned in free listing. Practical knowledge was analysed using a binary variable that takes a value of 1 when the number of plants recognised was >14.00, which was half of the plants included in the plant identification test. The number of plants known/recognised was assumed to be a suitable proxy for knowledge/skills. Coefficients of correlation between the applied variables were also calculated, but only the most relevant statistically significant correlations are mentioned in the results section.

**Table 1:** **Variables considered in the study and studies investigating these variables**

**Variable**	**Categories**	**Specification**	**Reference(s)**
Gender	Male/Female	-	[3,36,37,41]
Age	Age classes: 1 (7–13 yr); 2 (14–20 yr); 3 (21–30 yr); 4 (31–40 yr); 5 (>40 yr).	-	[17,24,41]
Age × Gender		Interaction factor	[7,11]
Refugee	Yes/No	Did the informant live in the local mountains as a refugee?	Not previously examined
Visit mountains	Yes/No	Does the informant visit the mountains in daily life?	In relation to occupation [17,41]
Work in the field	Yes/No	Does the informant plant, weed or harvest field crops?	In relation to occupation [17,41]
Place of residence	Arena Blanca/Pamuy	Where does the informant live?	[17,40]
Residence time	< or > than the average	How long has the informant lived in the area?	[41]
Main source of Knowledge	Grandparents; Parents; Other (school, self-taught)	From whom did the informant obtain knowledge of WEPs?	[19,23,24]
Distribution of the plant	Arena Blanca; Pamuy; Mountain; Milpa; Homegarden; Riverbank	Is the plant found nearby populated areas (Arena Blanca and/or Pamuy), in farmlands (milpa) or in other ecosystems (mountains, riverbank)?	[17,39,40]

The perception of how the knowledge was transmitted was analysed using the main source of knowledge about WEPs reported by each informant. The number of times that a source of knowledge was mentioned was counted and grouped by age class. Information regarding this topic from the semi-structured interviews was summarised and translated into English. This study initially attempted to assess the loss of knowledge by observing changes in recorded WEP knowledge across age. However, due to the difficulty of estimating such trends without a follow up study this research question was omitted.

## Results

### Wild edible plants of Río Negro

Forty-four WEP species belonging to 26 families were recorded in the study area. Table 2 provides detailed information about the plant species. Two taxa could not be identified, the herb *Tzimajuy* and the vine *Bejuco del cerro* (local names). The family that was represented by the highest number of taxa was Solanaceae with five taxa. Amaranthaceae, Arecaceae, Cactaceae and Leguminosae were represented by three taxa each. Anacardiaceae, Apiaceae, and Rosaceae were represented by two taxa each and the rest of the families (39%) by only one. One third of the taxa were trees, one fourth was herbs, and smaller fractions were shrubs, cacti, vines, palms and epiphytes. According to the key informants, 50% of the WEPs were also cultivated in the region at the time of the study, meaning that the plants grew naturally in the region but were also being cultivated by the villagers. The species that were not cultivated were gathered from the wild (12) or not consumed anymore and referred to as famine foods. Medicinal edibles represented 29.5% of all WEPs.

**Table 2:** **List of wild edible plants reported by the Río Negro inhabitants and associated characteristics**

**Spanish name**	**Achí name**	**Family**	**Species**	**Growth form**	**Part used**	**Preparation mode**	**Cultivated in the region?**	**Disease**	**Used part for medicinal purposes**	**Recommendations**	**Times mentioned in free list**	**Times identified**
Aguacate de mono	Roj’koy	Lauraceae	*Persea donnell-smithii* Mez.	Tree	Fruit*	Raw	Not cultivated, but a domesticated relative				4	36
Amaranto/Bledo	Labises	Amaranthaceae	*Amaranthus cf. hybridus* L.	Herb	Leaves and stem/Seed	Broth; Fried; Toast seeds (*poporopo*) to make a beverage (*atol*) or use them as cereals	Cultivated	Memory	Leaves	Include it in diet	24	43
Anona de montaña	Pak K’ewex	Annonaceae	*Annona reticulata* L.	Tree	Fruit	Raw	Cultivated				21	N/A
Apazote	Zikij	Amaranthaceae	*Dysphania ambrosioides* (L.) Mosyakin & Clemants*.*	Herb	Leaves and stem	Boil it in fish broth or with tomato; cook it with maize to make *tortillas*	Cultivated	Against amoeba and pinworms	Leaves	Grind and put it over the stomach with a cloth	16	46
Bejuco del cerro	Ukush Qachuu/Ixim	-	*-*	Vine	Root*	Grind and mix with maize (if any) to make *tortillas*; or peel it, chop it and boil it with the grinded maize and make *pixtones* (thick and smaller *tortillas*)	Not cultivated				7	N/A
Cabeza de viejo	Mam/K’mam	Cactaceae	*Cephalocereus maxonii* Rose (unresolved name)	Cacti	Fruit	Raw	Not cultivated				1	N/A
Capulín	Chapúl	Simaroubaceae	*Simarouba amara* Aubl.	Tree	Fruit*	Raw	Not cultivated				2	N/A
Caulote silvestre	Xuyuy	Malvaceae	*Guazuma ulmifolia* Lam.	Tree	Fruit*	Raw (now they do not eat it, it is mainly used to feed animals)	Cultivated	Infections	Leaves	Boiled	3	13
Chico zapote	Mu’y	Sapotaceae	*Manilkara zapota* (L.) P. Royen	Tree	Fruit	Raw	Not cultivated				29	57
Chilpepe	Rachaj’chó	Solanaceae	*Capsicum annuum* L.	Herb	Fruit	Raw	Cultivated	Gastritis	Fruit	Eat 7 of them every morning for 2 days, raw	2	N/A
Chipilín colorado	Muuch’	Leguminosae	*Crotalaria sagittalis* L.	Shrub	Leaves and stem	In broth or in *tamal*	Cultivated	Low pressure	Leaves and tender stem	Include it in diet	19	45
Cilantro silvestre	Culantó	Apiaceae	*Eryngium foetidum* L.	Herb	Leaves	Used as a condiment for broths, chicken and tomato sauce	Cultivated	Headache	Leaves	Include it in diet	4	52
Coyol	Map	Arecaceae	*Acrocomia aculeata* (Jacq.) Lodd. ex Mart.	Palm	Fruit	Peel fruit and suck. The seeds are also edible. Boiled with *panela* (sugar); or raw	Not cultivated				13	12
Guayaba de montaña	Cham Kaq’	Myrtaceae	*Psidium guajava* L.	Tree	Fruit	Raw	Cultivated	Diarrhoea and stomach ache	Young leaves	Tea and bath infusion	18	7
Huilihuiste	Huilihuiste	Rhamnaceae	*Karwinskia calderonii* Standl.	Tree	Fruit	Raw	Not cultivated				4	N/A
Izote	Pal’ki	Asparagaceae	*Yucca elephantipes* Regel	Tree	Flower	Boil first and then eat it in *tamal*; with egg as *torta* (omelette), or with chicken/hen	Cultivated				7	58
Jocote silvestre	Q’enum	Anacardiaceae	*Spondias purpurea* L*.*	Tree	Fruit and root*	Fruit eaten raw; root eaten raw (sweet)	Cultivated	Fever	Leaves	Drink it in tea together with guayaba and lemon leaves	35	61
Lechuga de Monte/Lechuguilla/Hierba de gallo	Rojob’ak	Apiaceae	*Eryngium ghiesbreghtii* Decne.	Herb	Leaves	Raw	Not cultivated				11	N/A
Loroco	Doroco	Apocynaceae	*Fernaldia pandurata* (A.DC.) Woodson	Vine	Flower	In *tamal* or with chicken	Cultivated				5	N/A
Macuy	Imu’t	Solanaceae	*Solanum americanum* Mill*.*	Herb	Leaves and stem	Broth; raw; with fish	Cultivated	Lung pain	Leaves	Grind and put in a cloth, leave it overnight to oxygenate and then put cloth on the back	29	38
Madre cacao	Reti cacó	Leguminosae	*Gliricidia sepium* (Jacq.) Walp.	Tree	Flower	Prepare it with *pepita* (ground pumpkin seeds)	Cultivated				2	N/A
Manzanita de montaña	Manzan de jullú	Rosaceae	*Malus pumila* Mill*.*	Tree	Fruit	Raw	Not cultivated				24	N/A
Mango	Mang	Anacardiaceae	*Mangifera indica* L.	Tree	Fruit	Raw	Cultivated				11	N/A
Miltomate	Po’a pix	Solanaceae	*Physalis philadelphica* Lam*.*	Herb	Fruit	Raw	Cultivated				9	53
Mora	Tukaan	Rosaceae	*Rubus adenotrichos* Schltdl*.*	Shrub	Fruit	Raw	Not cultivated				6	N/A
Nance	Tapa’l	Malpighiaceae	*Byrsonima crassifolia* (L.) Kunth	Tree	Fruit	Raw	Not cultivated				29	49
Nopal/Tuna	Ch’uuj	Cactaceae	*Opuntia spp.*	Cacti	Fruit	Raw	Cultivated	Gastritis, wounds	Leaves	Eat flesh of leaves; or apply flesh on wounds	3	37
Oreja de burro	Jotzotz	Amaranthaceae	*Iresine calea* (Ibafiez) Standl*.*	Herb	Leaves and stem	Prepare with *pepita* or slightly boiled	Not cultivated				2	N/A
Palma blanca	Pa’l	Arecaceae	*Brahea calcarea* Liebm*.*	Palm	Fruit and heart*	Cut the head of the palm, and take out the tender part (heart). Boiled; roasted; raw. Sour taste. Fruit eaten raw	Not cultivated				18	62
Palma suyate/Palma colorada	Suyate	Arecaceae	*Brahea dulcis* (Kunth) Mart.	Palm	Fruit and heart*	Cut the head of the palm, and take out the tender part (heart). Boiled; roasted; raw. Sour taste. Fruit eaten raw	Not cultivated				10	N/A
Palo de moco	Moco	Actinidiaceae	*Saurauia kegeliana* Schltdl*.*	Tree	Fruit	Raw	Not cultivated				4	N/A
Palo de pito	Pipí	Leguminosae	*Erythrina berteroana* Urb*.*	Tree	Flower and young leaves	Prepare the flowers with *pepita*; the leaves boiled (to avoid somnolence it has to be boiled three times)	Cultivated	Insomnia	Flower and young leaves	Prepare the flowers with *pepita*; the leaves boiled	5	57
Palo ramón/Ujushte	Ash	Moraceae	*Brosimum alicastrum* Sw*.*	Tree	Seed	Boil and mix with maize to make *tortillas*; with egg to make a *torta* (omelette); or make a beverage (*atol*)	Not cultivated				11	3
Papaya de montaña	Papaisis	Caricaceae	Vasconcellea cauliflora (Jacq.) A.DC.	Tree	Fruit	Raw	Cultivated				6	N/A
Pata paloma	Rej’tzi	Phytolaccaceae	*Phytolacca icosandra* L.	Shrub	Leaves and stem	Raw or in *pepita*	Not cultivated				3	N/A
Piñuela	Ratí chumil	Bromeliaceae	*Bromelia alsodes* H. St. John	Epiphytic	Heart*	Raw	Not cultivated				1	N/A
Piñuela roja	Tz’op	Bromeliaceae	*Bromelia pinguin* L.	Epiphytic	Fruit	Raw	Not cultivated				1	N/A
Pitaya o Pitahaya	Pitahay	Cactaceae	*Hylocereus undatus* (Haw.) Britton & Rose	Cacti	Fruit	Raw	Cultivated				11	44
Quequesque	Tup	Araceae	*Xanthosoma robustum* Schott.	Herb	Young leaves	Broth or in *tamal*	Cultivated				5	42
Quixtán	Quixtan	Solanaceae	*Solanum wendlandii* Hook*.*	Vine	Leaves	Boiled	Cultivated				1	N/A
Tomatillo	Iximpix	Solanaceae	*-*	Herb	Fruit	Raw	Cultivated	Skin swelling	Leaves	Grind and put it on the skin with a cloth	0	N/A
-	Tzimajuy	-	*-*	Herb	Leaves and stem	Boiled	Not cultivated				9	N/A
Tushiboy/Hierba de iboy	Tushiboy	Lamiaceae	*-*	Shrub	Leaves and stem	Prepare it with *pepita*; or together with maize in *tortillas*; or in broth	Not cultivated				3	N/A
Verdolaga	Paxlaq	Portulacaceae	*Portulaca oleracea* L*.*	Herb	Leaves and stem	Boiled	Not cultivated	Memory, sight, anaemia	Leaves and tender stem	Boiled and eaten with *tortillas*	15	50

Underlined names: medicinal edibles (*): famine foods. N/A refers to the plants that were not included in the plant identification test.

Most of the WEPs could be found in the surrounding mountains (37) and around the fields (*milpa*) (28), 11 species were found in the vicinity of the subvillage Pamuy, while only two were found in the vicinity of the other subvillage, Arena Blanca. Ten taxa grew wild on the riverbanks and nine were found in homegardens. Four of the nine taxa found in the homegardens could be cultivated.

**Figure 2:**
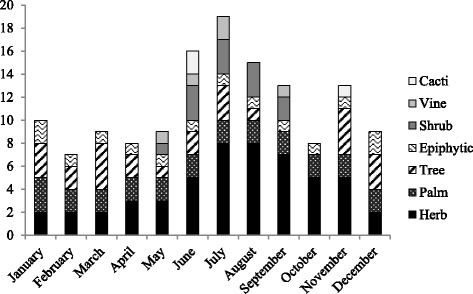
Total number of WEPs available per month by plant growth form. Individual species can be available in several months. Abundance is not taken into account.

Of the 44 plant species, 25 had edible fruit, 14 edible leaves, nine tender edible stems, four edible flowers, three edible palm hearts, two edible roots and two edible seeds. Men and children were the main collectors of most edible plants, gathering mainly fruits, palms and edible parts from tall trees, while women collected herbs and edible parts from bushes and small trees. Only five plants were reported to be available throughout the year. Edible plants could be found throughout the year but with greater availability from June to November (Figure 2).

### Individual traditional knowledge of wild edible plants

#### Knowledge transmission

**Figure 3:**
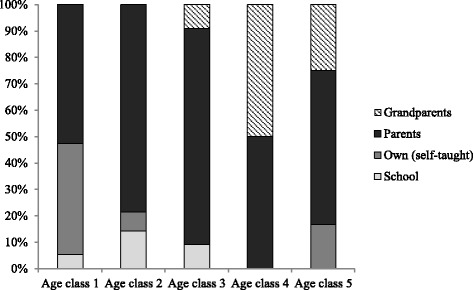
Distribution of the main sources of general knowledge by age classes. Age Classes: 1 (7–13 yr); 2 (14–20 yr); 3 (21–30 yr); 4 (31–40 yr); 5 (>40 yr).

Generally knowledge of WEPs was reported to be transmitted from relatives (grandparents and parents) or from school, and some people claimed to have learned about WEPs on their own (self-taught). In 76% of the cases, the knowledge was transmitted from relatives. The distribution of the main sources of knowledge by age classes is shown in Figure 3. Parents were the most important source of knowledge for young people, while grandparents were more important for older age classes.

Informants that had lived in the mountains as refugees (age classes 4 and 5) explained that their grandparents knew what wild plants could be used as food, because they suffered a previous period of hunger. People that did not spend the years in the mountains with their grandparents explained that they used the trial and error method. Some informants lived with their mother or father only and reported her/him as the main source of knowledge. Other informants that were born after the times of refuge explained that the knowledge was transmitted visually and orally when their mother or grandmother was cooking. Basically, they had learned about the plants because the plants were eaten.

The majority of the informants mentioned less than the average number of plants mentioned in the free listing (7.15) (68%) but identified more than 50% of the plants in the plant identification test (79%). Almost all informants (86%) that had acquired their knowledge from grandparents mentioned and identified more than 50% of the WEPs. Conversely, the majority of informants who had acquired their knowledge from other sources than the grandparents listed less than the average number of plants mentioned (67%). Especially informants who were self-taught listed and identified less plants (Table 3).

**Table 3:** **Impact of main source of theoretical and practical knowledge**

**Main source of knowledge**	**Theoretical knowledge**		**Practical knowledge**	
	**WEPs mentioned**		**WEPs recognised**	
	**≤7.15**	**>7.15**	**≤14.00**	**>14.00**
Grandparents (11%)	14%	86%	0%	100%
Parents (65%)	73%	28%	18%	83%
School (6%)	50%	50%	0%	100%
Own (self-taught) (18%)	91%	9%	55%	45%
**Total**	**68%**	**32%**	**21%**	**79%**

Percentage of informants, categorised by self-reported main source of knowledge (left column), that mentioned more or less than the average number of plants (7.15) mentioned in the free listing (middle column) and number of informants that recognised more or less than half of the plants (28/2 = 14) in the plant identification test (right column).

#### Knowledge distribution and factors determining it

**Figure 4:**
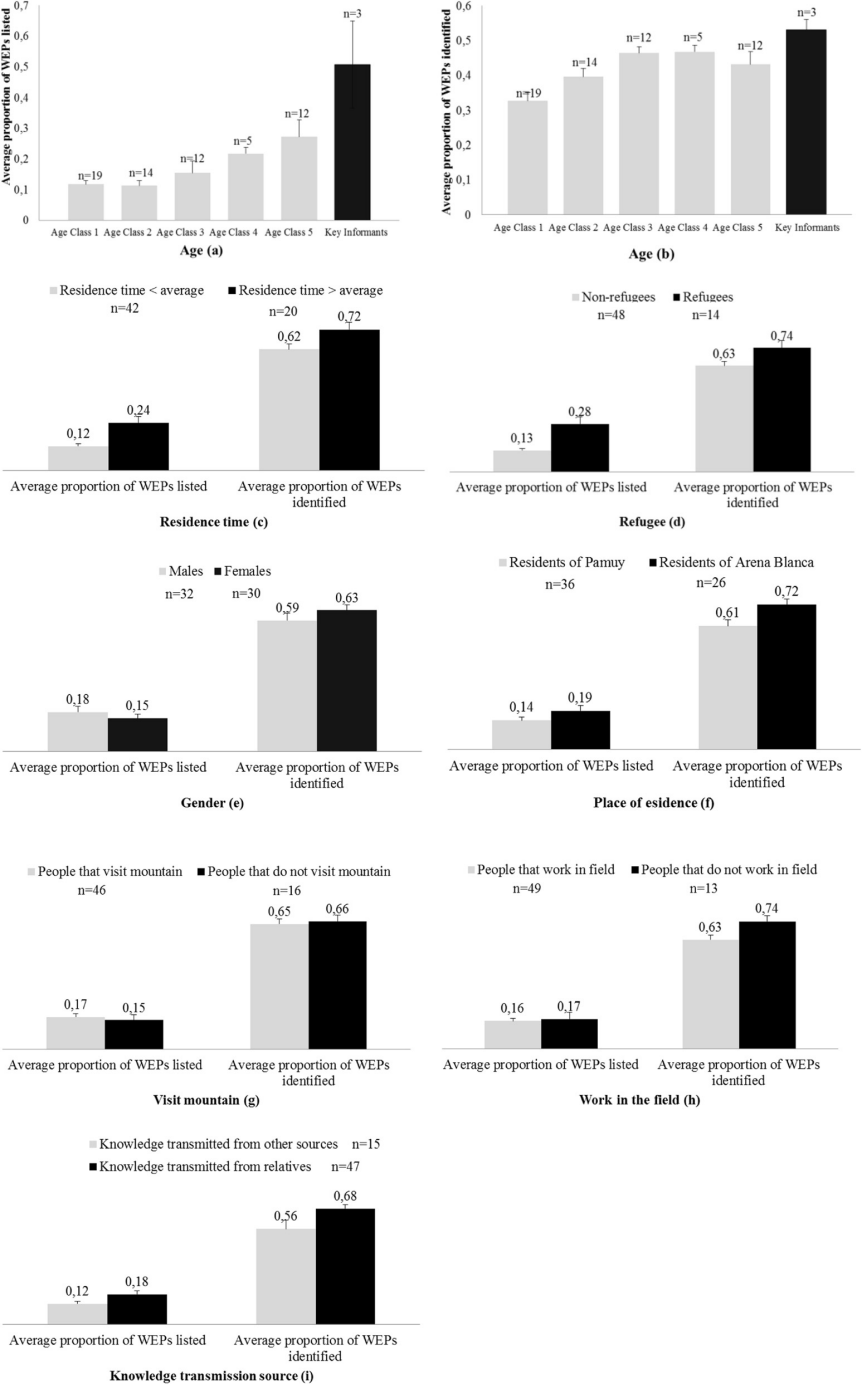
Average proportions of WEPs mentioned in the free listing method and identified in the plant identification test by category. Categories are: age classes and key informants **(a, b)**, residence time **(c)**, refugee factor **(d)**, gender **(e)**, residence place **(f)**, visit mountain factor **(g)**, work in fields **(h)** and main source of knowledge **(i)**; error bars indicate standard error of the mean.

The average number of plants listed by the informants was 7.15 ± 0.69 (n = 62; mean ± standard error SE). However, the key informants’ group listed on average 22.33 ± 6.23 plants (n = 3, mean ± SE), more than three times as many as the rest of the population on average (6.37 ± 0.50; n = 59, mean ± SE). The average number of plants identified was 17.65 ± 0.60 (n = 62; mean ± SE), i.e. 65% of the plants presented to each informant. The difference between the groups with regard to number of plants identified correctly was smaller than with regard to number of plants listed, key informants recognising 23 on average (23.33 ± 1.33; n = 3, mean ± SE) and the rest of the population recognising 17 plants on average (17.36 ± 0.61; n = 59, mean ± SE). The number of plants listed increased across age classes (Figure 4a). By contrast the average number of plants identified showed only slight variation across age classes with a slightly higher proportion of plants being identified by older age groups (age classes 3–5) than by younger age groups (age classes 1–2) (Figure 4b). To some extent the ‘age’ variable also represented the variable ‘refugee’ (r = 0.73, Prob > |r| < .0001) and ‘residence time’ (r = 0.87, Prob > |r| < .0001) as these were highly correlated with age. In fact, the people that were refugees were old enough to have lived during the conflict and had stayed in the area for longer time than others. Therefore, these groups also demonstrated higher theoretical and practical knowledge on average (Figure 4c, d). The numbers of plants listed and identified were similar between genders, but male informants listed more plants on average than females, whereas female informants identified a slightly higher proportion of plants than males (Figure 4e). The residents of Arena Blanca listed and identified more plants on average than the residents of Pamuy (Figure 4f). Informants who stated that they visited the surrounding mountains regularly and those who did not performed equally well on average in both tests (Figure 4g). People who did not work in the fields recognised more plants on average than people who did, but no difference was found with respect to the average number of plants listed (Figure 4h). On average informants who received their knowledge about WEP from relatives listed and identified more plants than informants who received knowledge from other sources (Figure 4i).

Based on the Chi-square independence tests it appears that theoretical knowledge, i.e. listing more/less plants than the average informant, depended on residence period, having lived in the mountains as a refugee (refugee factor), age, the age × gender interaction factor and the main source of knowledge about wild edible plants (Table 4). Practical knowledge, i.e. the skill to identify more/less than half of the plants included in the test, appeared to depend on some of the same variables as theoretical knowledge (age, main source of knowledge and gender × age) and was furthermore dependent on gender, visits to mountains, work in fields and residence place.

**Table 4:** **Results of Chi-square independence tests for two-way tables with theoretical and practical knowledge in columns and the two categories of the binary variables in rows**

	**Theoretical knowledge**		**Practical knowledge**	
**Variable**	**Chi-square**	**Pr > Chi** ^**2**^	**Chi-square**	**Pr > Chi** ^**2**^
Age class 1 (7–13 yr)	**3.4001**	**0.0652**	**7.3863**	**0.0066**
Age class 2 (14–20 yr)	2.6730	0.1021	0.4872	0.4852
Age class 3 (21–30 yr)	0.3587	0.5492	**3.9478**	**0.0469**
Age class 4 (31–40 yr)	**5.6727**	**0.0172**	1.4429	0.2297
Age class 5 (>40 yr)	**8.0620**	**0.0045**	0.1460	0.7024
Residence Period	**14.4839**	**0.0001**	0.6345	0.4257
Refugee Factor	**23.6472**	**<.0001**	0.4872	0.4852
Gender	0.8316	0.3618	**7.3863**	**0.0066**
Gender × Age	**19.2453**	**0.0232**	**15.8071**	**0.0710**
Residence Place	0.7886	0.3745	**4.7622**	**0.0291**
Visit Mountain	1.8007	0.1796	**2.8189**	**0.0932**
Work in the field	0.2897	0.5904	**4.3640**	**0.0367**
Main Source of Knowledge	**10.5996**	**0.0050**	**5.4262**	**0.0663**

Statistically significant (p < 0.1) results are shown in bold.

## Discussion

### Wild edible plants of Río Negro

Similar numbers of WEPs have been recorded in other studies with comparable climatic conditions. Two studies found 22 wild edible species [3] and 23 and 19 edible trees [52] in three communities located in subtropical moist forest of Guatemala. Ladio and Lozada [34] documented 42 WEPs in a dry forest of a plateau in north-western Patagonia, where 38 were still used, while Maldonado et al. [53] documented 56 in a seasonally dry tropical forest in the central part of southern Mexico. The most frequently consumed WEPs found in this study are trees, which is similar to results from other studies around the world [13,54,55], probably because trees frequently produce edible fruits that are highly valued by people [53]. By contrast, a review on plants used by indigenous groups in Mexico, including the Mayans, found that herbs were consumed more often than products from trees or shrubs [56]. This study agrees with several other studies showing that fruits are the most commonly used plant part [6,8,13,36,38,39,53,55], while in some East Asian studies the leaves were the most frequently used part [37] as were the young sprouts [54].

The most important plant families, relative to the other families and in terms of number of species [53] were: Solanaceae, Amaranthaceae and Leguminosae that have also been categorized as relevant sources of edible species in Mesoamerica by Bressani [57], Azurdia [27] and De Macvean and Pöll [58]. The Solanaceae is considered a family of high global importance [59].

About 50% of the WEPs reported in this study were also cultivated by the Achí Mayans. This is similar to the study by Blancas and colleagues [39] (45%). No other studies were found that investigated the proportion of reported WEPs plants also being cultivated. This is of importance as Mesoamerica has been one of the most active places in plant domestication in the world [60].

The percentage of medicinal edibles reported in this study (29.5%) is comparable to percentages observed by Ladio et al. [40] (35%) and Uprety et al. [38] (24%), while other studies reported twice as many [55] (69%) or did not find a clear role of medicinal edibles in the region [8]. The surroundings of the dwellings and nearby footpaths are normally preferred places of food plant gathering and therefore most WEPs are usually related to such areas [23,36]. This is contrary to the present study where most of the WEPs were found in the forest, quite far from the villages. The reason for the unusually high frequency of forest food plants is probably historical and linked to the period of extreme dependency on WEPs during the refuge in the mountains. Hence, ex-refugees also reported most WEPs. Furthermore, the dam flooded the fertile farm land previously inhabited by the communities. The lack of WEPs in the vicinity of the dwellings may be a consequence of the infertile and rocky land where the communities re-settled after the construction of the dam.

### Individual traditional knowledge of wild edible plants

#### Quantification of knowledge

The population under study turned out to be rather heterogeneous with regard to theoretical knowledge, while practical knowledge was more evenly distributed. This might be because the spectrum of possible answers in the free listing method was higher than for the plant identification test. Free listing is a more demanding exercise, requiring the informant to patiently and systematically go through and explain his/her knowledge. Informants might in fact know more plant species than they list [61]. This is supported by the fact that the average proportion of plants identified is higher than the average proportion of plants mentioned. Ladio and Lozada [34] found contrasting results, although instead of using plant identification they used reports of consumed plants. In our study, the low proportions of plants listed by informants is a consequence of the fact that the proportions were calculated using the plant list created with the key informants as reference, which was assumed to include all the WEPs that can be found in the area. However, nobody mentioned all of the 44 plants; the highest number recorded was 30 plants. Nevertheless, when identifying plants that people actually use free listings are believed to produce a more accurate result than identification tests [61]. Other studies indicate that combining the free listing method with other methods such as a questionnaire survey is helpful to cover significant information regarding the plants under study [8].

Some families were represented in the study by only one person, for instance in cases where a single mother was living with two children that were not old enough to participate in the study. This made it impossible to calculate the variation of knowledge within and between families and to take into account autocorrelation between family members.

The results obtained might have been different if temporary migrants had been present at the time of the study, as they were likely to have other occupations and/or higher level of education and experience from other environments. Ohmagari and Berkes [20] concluded that schooling in a foreign country limited the acquisition of original skills and knowledge.

#### Knowledge acquisition, transmission and loss

WEP knowledge is mainly perceived to be transmitted from relatives and, in accordance with our first hypothesis, the source of knowledge does have an influence on theoretical and practical knowledge. The younger generation receives knowledge consciously from school, and systematically from everyday life, from helping their parents in the fields, going to the mountains to gather wood, cooking, or when having their meals. The older generation (>40 years old) did not attend school and neither did many of the women born before 1991. They received knowledge about natural resources from their parents, following the stages of knowledge acquisition presented by Ohmagari and Berkes [20] and Zarger [21]: familiarization, observation and helping. Formal education has been reported to be negatively correlated with traditional knowledge [20] and this study agrees with this statement. The transmission through relatives is essential to maintain knowledge and use of wild edible resources.

#### Knowledge of WEPs

The average number of plants listed by the informants was slightly lower than the numbers obtained in the study by Maldonado and colleagues [53] (10.7 ± 1.53, mean ± SD). The elder and ex-refugees showed higher theoretical knowledge than others, thus supporting our second and third hypotheses. The effect of age has been explained by Araújo and Lopes [41], stating that living in a certain environment for longer time increases the chances of using a resource and thus accumulating knowledge of local plants. The practical knowledge, or skills to identify plants, increase with age and showed a decline in the late phase of life, as explained by Zarger and Stepp [24], Reyes-García et al. [17] and Araújo and Lopes [41]. In addition, the times of refuge and the necessity to eat certain plants that are not usually preferred may have augmented the difference in knowledge between age classes, as stated in the third hypothesis.

Gender did not appear to influence the total number of plants listed. Therefore, the fourth hypothesis stating that women are more knowledgeable than men is not supported by the survey. This is in accordance with studies by González et al. [36] and Ghorbani et al. [37], where no or small differences were found. However, gender did appear to influence practical plant identification skills, as females identified more plants on average than men, probably because they prepare the meals. This agrees with the findings of Nesheim et al. [3] and Araújo and Lopes [41], who found that men knew more plant species used in construction, whereas women knew more domestic use plants, such as edible and medicinal plants.

In relation to the fifth hypothesis, this study found that number of plants known and identified depends on the gender × age interaction factor. This is in agreement with previous studies, where older women were the most knowledgeable group regarding food plants [7,11], although other studies have found no relation [37] or marginal significance only [36].

Regarding the sixth hypothesis, residents of Pamuy were expected to possess greater knowledge and skills than residents of Arena Blanca as abundance of WEPs was higher and distance to fields, riverbanks and mountains was lower at Pamuy [17,40]. The residence place was shown to influence the number of plants recognised, but contrary to expectations the residents of Arena Blanca identified the highest proportion of plants.

When people maintain a close relation to natural resources their practical skills are unlikely to decline [17,41]. Therefore, as stated in the seventh hypothesis, it was expected that people who work in the fields and visit the mountains would know and recognise more plants than people who do not. Furthermore, most of the WEPs are found in these places so the chances of encountering them are higher. No difference was found in the free listing results but surprisingly, it emerged that informants who do not frequent these places identified more plants. A possible partial reason may be that the plants used in the test were common, as they are used in everyday diets and can be found in markets of other areas. If more famine foods or site-specific plants were included the results would presumably be different.

These results contribute evidence on how WEP knowledge distribution vary between the populations of different societies, where differences in social structures, history and culture shape how the knowledge is acquired, transmitted and maintained [19].

## Conclusions

The survey and use of wild edible plants of Río Negro serves as a reference point and as a useful compilation of knowledge of the community for present and future generations.

Knowledge of wild edible plants was found to be mainly transmitted through relatives. Informants who acquired knowledge from relatives knew more plants and developed better skills to recognise plants than informants taught in school or who acquired knowledge on their own.

The main factor influencing the distribution of theoretical and practical knowledge was the historical relation to the surrounding environment. Río Negro has been affected strongly by the building of the Chixoy dam, where relatives, houses and fertile lands were lost, inhabitants persecuted and the survivors were forced to take refuge in the mountains for several years. The extreme dependency on WEPs during the years of refuge by some of the elders of the community resulted in an uneven distribution of theoretical knowledge among the population. Practical knowledge was found to be more homogenously distributed at least for commonly used WEPs. The community is still partially isolated and most fertile lands were flooded by the Chixoy dam. Hence, traditional knowledge on WEPs is still important to the inhabitants though some WEPs are not used any longer. This study shows that traditional ecological knowledge is determined by the specific ecological, historical, cultural and socio-economic context of the population under study.
